# Recurrent Somatic Copy Number Alterations and Their Association with Oncogene Expression Levels in High-Grade Ovarian Serous Carcinoma

**DOI:** 10.3390/life13112192

**Published:** 2023-11-10

**Authors:** Hillary P. Esplen, Richard K. Yang, Awdhesh Kalia, Zhenya Tang, Guilin Tang, L. Jeffrey Medeiros, Gokce A. Toruner

**Affiliations:** 1 Graduate Program in Diagnostic Genetics and Genomics, School of Health Professions, The University of Texas MD Anderson Cancer Center, Houston, TX 77030-4009, USA; 2 Department of Pathology, The University of Texas MD Anderson Cancer Center, Houston, TX 77030-4009, USA; 3 Department of Hematopathology, The University of Texas MD Anderson Cancer Center, 6565 MD Anderson Avenue, Houston, TX 77030-4009, USA; 4 Department of Pathology and Microbiology, College of Medicine, University of Nebraska Medical Center, Omaha, NE 68198-7815, USA

**Keywords:** high-grade ovarian serous carcinoma, somatic copy number aberration, gene expression

## Abstract

Somatic copy number alterations (SCNAs) are frequently observed in high-grade ovarian serous carcinoma (HGOSC). However, their impact on gene expression levels has not been systematically assessed. In this study, we explored the relationship between recurrent SCNA and gene expression using The Cancer Genome Atlas Pan Cancer dataset (OSC, TCGA, PanCancer Atlas) to identify cancer-related genes in HGOSC. We then investigated any association between highly correlated cancer genes and clinicopathological parameters, including age of diagnosis, disease stage, overall survival (OS), and progression-free survival (PFS). A total of 772 genes with recurrent SCNAs were observed. SCNA and mRNA expression levels were highly correlated for 274 genes; 24 genes were classified as a Tier 1 gene in the Cancer Gene Census in the Catalogue of Somatic Mutations in Cancer (CGC-COSMIC). Of these, 11 Tier 1 genes had highly correlated SCNA and mRNA expression levels: *TBL1XR1*, *PIK3CA*, *UBR5*, *EIF3E*, *RAD21*, *EXT1*, *RECQL4*, *KRAS*, *PRKACA*, *BRD4*, and *TPM4*. There was no association between gene amplification and disease stage or PFS. *EIF3E*, *RAD21*, and *EXT1* were more frequently amplified in younger patients, specifically those under the age of 55 years. Patients with tumors carrying *PRKACA*, *BRD4*, or *TPM4* amplification were associated with a significantly shorter OS. *RECQL4* amplification was more frequent in younger patients, and tumors with this amplification were associated with a significantly better OS.

## 1. Introduction

Ovarian cancer ranks as the eighth most prevalent cancer among women worldwide [1]. In the United States, it stands as the second most common gynecological cancer, with an estimated 19,710 new cases and 13,270 deaths projected for 2023 [2]. Ovarian cancer is also the fifth leading cause of cancer death among women over 40 years [2]. Five-year survival rates vary substantially and correlate with the stage at diagnosis: 93% for patients with localized disease, 74% for those with regional disease, and 31% for those diagnosed at a distant stage, leading to an overall five-year survival rate of 50% [2].

Ovarian cancer can be categorized into subtypes based on its cellular origin, including epithelial cells, germ cells, and stromal cells [3]. Of these, epithelial ovarian tumors are the predominant type of malignant ovarian cancer, accounting for over 90% of cases [4]. Within the epithelial category, tumors can be further classified based on their histological features. Serous carcinomas emerge as the most common subtype, further divided into high-grade (HG) and low-grade (LG) tumors. Notably, high-grade serous ovarian carcinoma (HGOSC) stands out as the most frequently diagnosed subtype within epithelial ovarian cancers.

In 2020, the World Health Organization (WHO) released an updated classification distinguishing between high-grade serous ovarian carcinoma (HGOSC) and low-grade serous ovarian carcinoma (LGOSC). One histopathological distinction between these serous subtypes is the variation in nuclear size; HGOSC displays a variation greater than three-fold and high mitotic activity, whereas LGOSC shows less variation in nuclear size and lower mitotic activity [5]. LGOSC may present mutations involving *BRAF, EIF1AX*, *KRAS*, *NRAS*, and *USP9X*, and often exhibits copy number alterations, with the most prevalent being the loss of chromosome 9p and homozygous deletions at the *CDKN2A/B* locus [6]. Conversely, HGOSC is associated with several germline mutations, including *BRCA1*, *BRCA2*, *BRIP1,* and *RAD51D* [7]. HGOSC tumors typically harbor a *TP53* mutation and exhibit high level somatic copy number aberrations (SCNAs), which are associated with significant chromosomal instability [8]. Previous studies have identified amplifications of loci on chromosome 19 in HGOSC [9,10,11]. Notably, *BRD4* has emerged as a therapeutic target, leading to the development of various bromodomain and extra terminal domain inhibitors (BETi) [12,13].

Beyond the amplifications on chromosome 19, other chromosomal regions also exhibit frequent copy number changes. Several studies have highlighted the amplification of known oncogenes, such as *MECOM* (3q26.2) and *MYC* (8q24) [14,15,16]. Other regions, including chromosomes 1, 5, and 12, have shown frequent amplifications at loci of oncogenes like *MYCL1* (1p34.2), *TERT* (5p13.33), and *KRAS* (12p12.1) [14,16]. Additionally, Engler et al. [16] reported significant deletions in over 70% of HGOSC tumor samples, specifically in chromosomal regions 16q24.2 and 22q13.33. Furthermore, homozygous deletions of tumor suppressor genes such as *PTEN* (10q23.21), *RB1* (13q14), and *NF1* (17q11.2) in a subset of tumor samples were identified [17]. In summary, regions with frequent copy number changes, housing various oncogenes and tumor suppressor genes, have been closely linked with HGOSC.

Identifying recurrent SCNAs in cancer allows researchers to delve deeper into the abnormalities propelling cancer development and progression. However, the presence of SCNAs in a tumor can sometimes introduce complexities. Challenges arise when multiple candidate genes exist within a single affected region, especially in tumors exhibiting a high level of copy number changes [18]. Assessing whether there is also an alteration in gene expression can provide insights, as copy number changes do not always result in altered gene expression. HGOSC is an example of a neoplasm characterized by a very high level of copy number changes, averaging around 10 events per case [19]. Pinpointing oncogenes and tumor suppressor genes that exhibit both recurrent SCNAs and alterations in gene expression in HGOSC could yield valuable insights into its pathogenesis.

We hypothesized that frequently observed somatic copy number alterations (SCNAs) play a crucial role in the pathogenesis of high-grade serous ovarian cancer (HGOSC) through altering the expression levels of critical cancer-associated genes. The primary aim of this study was to identify cancer-associated genes previously implicated in a range of human cancers but not yet associated with HGOSC. The secondary aim was to correlate identified genes with recurrent SCNAs to clinicopathological parameters, including age at diagnosis, disease stage, overall survival, and progression-free survival. To accomplish these aims, we used the TCGA Pan Cancer Atlas OSC dataset, accessed through cBioPortal, to examine the correlation between the copy number changes of genes with recurrent SCNAs and gene expression levels. We then used the Tier 1 Cancer Gene Census in the Catalogue of Somatic Mutations in Cancer (CGC-COSMIC) to identify cancer-related genes in HGOSC.

## 2. Material and Methods

### 2.1. TCGA, PanCancer Atlas High-Grade Ovarian Serous Carcinoma Profiled Samples

HGOSC profiled samples were identified in the publicly available TCGA, Pan Cancer Atlas dataset (Ovarian Serous Cystadenocarcinoma (TCGA, PanCancer Atlas) [20]) from cBioPortal for Cancer Genomics [21,22].

To be included in the study, profiled samples were required to meet two criteria. First, each sample had to have both CNA gene and mRNA expression data. Second, the tumor was classified as a grade 3 or had a *TP53* mutation. Clinical data for each profiled sample were retrieved through tools available within cBioPortal. The publicly accessible data portal, cBioPortal “https://www.cbioportal.org (accessed on 5 May 2023)”, provides genomic information including DNA copy-number data, mRNA expression data, gene mutation, and protein-level data of various cancer types. De-identified patient clinical data, such as age at diagnosis, tumor stage, OS and PFS status, and types of treatments received, also can be accessed [21,22].

The TCGA sample collection process has been previously described in detail [20]. In brief, partnering tissue source facilities obtained samples of tumor tissue, proximal normal tissue, and normal whole blood as well as clinical data from each patient. Board-certified pathologists reviewed tissue samples to confirm the histopathological diagnosis using the edition of the World Health Organization (WHO)/International Agency for Research on Cancer (IARC) Classification of Tumors that was current at the time accessioning the samples [20].

Two hundred and seventy-two profiled samples met the criteria for inclusion in this study (Supplementary Table S1). The stages of these neoplasms were as follows: 1 stage I, 16 stage II, 220 stage III, 33 stage IV, and 2 with undocumented disease stage. The median patient age was 58 years (range, 30–87).

### 2.2. Identification of Genes with Recurrent Somatic Copy Number Alterations

The copy number status of each gene and the methodology were provided in detail previously [20]. To summarize, an Affymetrix Single Nucleotide Polymorphism (SNP) 6.0 array containing more than 940,000 probes to detect copy number variation was used to obtain data for chromosomal gains and losses [23]. Copy number gains and losses at the gene level were then determined using the Genomic Identification of Significant Targets in Cancer 2.0 (GISTIC 2.0) analysis. The statistical method, GISTIC 2.0, analyzed the frequency and amplitude of SCNAs to determine significant abnormal regions and the genes within those regions that possibly contributed to cancer development [24,25]. In cBioPortal, the copy number status of a gene within a tumor sample was further described as a deep or homozygous deletion, shallow deletion, diploid, gain, or amplification dependent on the thresholds set within GISTIC 2.0 [26]. 

The CNA gene dataset was extracted from cBioPortal and provided information on genes with detected SCNAs, the gene cytoband, SCNA classification (amplification or homozygous deletion), the number of profiled samples with the copy number change, and the frequency of SCNAs. Genes with SCNAs were filtered and identified as recurrent if the frequency, defined as the percentage of patients with SCNAs divided by the total number of profile patient samples, was at least 10% (≥0.10). GRCh38 coordinates of each gene were obtained through batch processing of genes in the University of California, Santa Cruz (UCSC) Table Browser through Galaxy “https://usegalaxy.org (accessed on 5 May 2023)”, a publicly accessible scientific analysis platform that provides tools for genomic analysis [27]. These coordinates were used to determine genomic blocks with recurrent SCNAs.

### 2.3. Correlation between Genes with Recurrent SCNA Correlation and mRNA Expression

To assess the impact of SCNAs on gene expression, we plotted log2 copy number values of genes with recurrent SCNAs against their mRNA expression z-scores, relative to diploid samples (RNA Seq V2 RSEM). The mRNA expression z-scores represent the deviation of a gene’s expression from the mean expression of diploid samples. We used Pearson correlation coefficients obtained from cBioPortal to gauge the strength of the linear relationship between the copy number alterations and gene expression levels. These coefficients range from −1 to +1, with a correlation deemed strong if the Pearson correlation coefficient was greater than or equal to 0.55 (r ≥ 0.55).

### 2.4. Identification of Cancer-Related Genes with Recurrent SCNA

We cross-referenced genes with recurrent SCNAs against the Tier 1 Cancer Gene Census (CGC) list within the Catalogue of Somatic Mutations in Cancer (COSMIC) (version 98, May 2023) to pinpoint cancer-related genes. COSMIC “https://cancer.sanger.ac.uk/cosmic (accessed on 5 May 2023)” compiles comprehensive information on genomic abnormalities and their roles in various cancers. The CGC project within COSMIC classifies genes into two tiers based on their evidence supporting a role in oncogenesis. Tier 1 genes are substantiated by at least two independent publications characterizing the somatic mutations and the biological processes driving cancer development [28,29]. Tier 2 genes, while implicated in cancer, have less extensive evidence regarding their mutation patterns and functional effects [28,29]. 

### 2.5. Association of Cancer Related Genes and Clinicopathological Parameters

Statistical analyses were conducted to determine the association of strongly correlated, Tier 1 CGC-COSMIC genes with recurrent SCNAs with different clinicopathological parameters using R software (v4.2.2). We used Fischer’s exact test for categorical variables such as age of diagnosis and disease stage, employing 55 years as the cutoff age based on the mean age of HGOSC patients being 65 years [5]. We classified tumor samples into early-stage (I and II) and late-stage (III and IV) cancer for the disease stage.

The associations between cancer-related genes and patient outcomes, including overall survival (OS) and progression-free survival (PFS), were evaluated using the Kaplan–Meier method. We utilized clinical information from the last follow-up to calculate OS and PFS. OS was measured from the date of initial diagnosis to the date of death, with living patients being censored in the analysis. PFS was defined as the time from initial diagnosis to the last clinical follow-up or documented disease progression for living patients. Survival curves were compared and analyzed using GraphPad Prism (v9.0.0), calculating *p*-values (Mantel–Cox test), median survival times for OS and PFS, and hazard ratios (HR) (Mantel–Haenszel method). We considered *p*-values less than 0.05 (*p* < 0.05) to be statistically significant.

## 3. Results

### 3.1. Genes with Recurrent SCNA

Seven hundred and sixty-nine genes with recurrent SCNAs were observed. Coordinates of these genes are presented in Supplementary Table S2. These genes were located at 14 genomic segments on chromosome arms 1p, 3q, 5q, 8p, 8q, 11q, 12p, 19p, 19q, and 22q. The sizes of these segments ranged from 0.25 Mb to 42.93 Mb. The SCNA segments located at 5q11.2-q12.1, 8p23.3-p23.2, and 22q13.32-q13.33 were deletions, and the remaining 11 segments located at 1p34.3-p34.2, 3q25.2-q29, 8q22.2-q23.2, 8q23.3-q24.23, 8q24.23-q24.3, 11q14.1-q14.1, 12p12.1-p11.23, 19p13.13-p13.11, 19p12-p12, 19q12-q12, and 19q13.2-q13.2 were amplifications (Table 1).

### 3.2. Highly Correlated, Tier 1 CGC-COSMIC Genes with Recurrent SCNA

**Figure 1:**
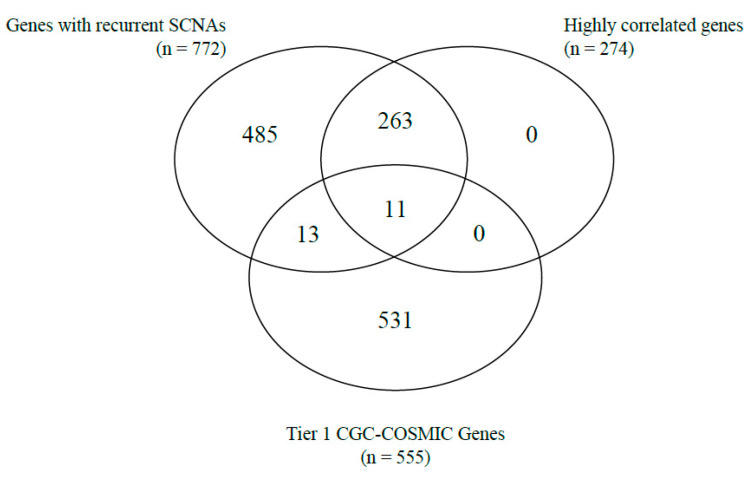
Venn-diagram of the number of genes with recurrent SCNA, highly correlated genes, and Tier 1 CGC-COSMIC genes.

**Figure 2:**
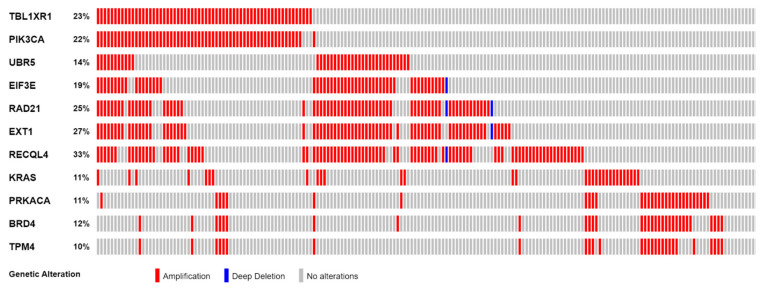
Frequency of SCNAs in 11 highly correlated Tier 1 CGC-COSMIC genes.

Correlations between gene copy number and gene expression, as well as the identification of cancer-related genes based on the Tier 1 CGC-COSMIC list, were performed among the genes with recurrent SCNAs. A total of 274 genes were highly correlated (Supplementary Table S2). Of the Tier 1 CGC-COSMIC gene list (Supplementary Table S3), 24 genes were identified as known cancer-related genes. Among these, 13 genes were not highly correlated, and 11 genes satisfied both criteria as highly correlated, Tier 1 CGC-COSMIC genes (Table 1, Figure 1 and Figure 2).

**Figure 3:**
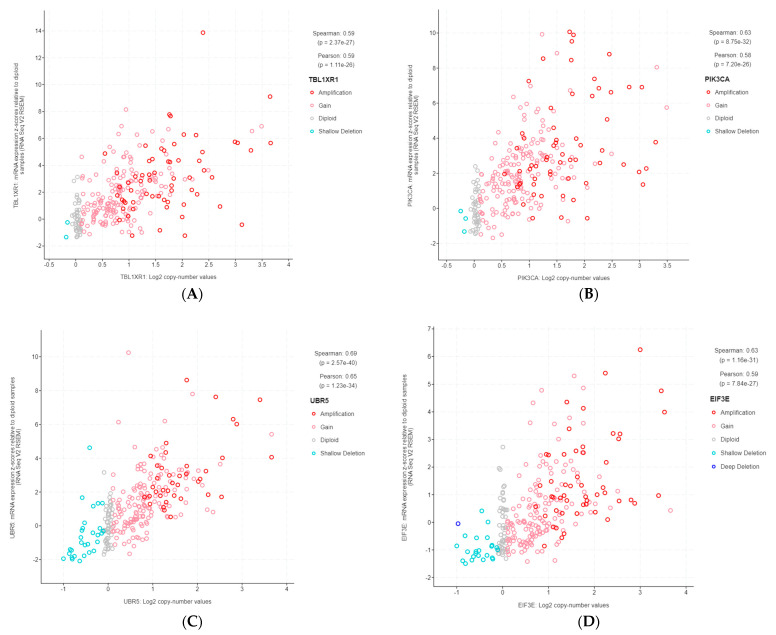

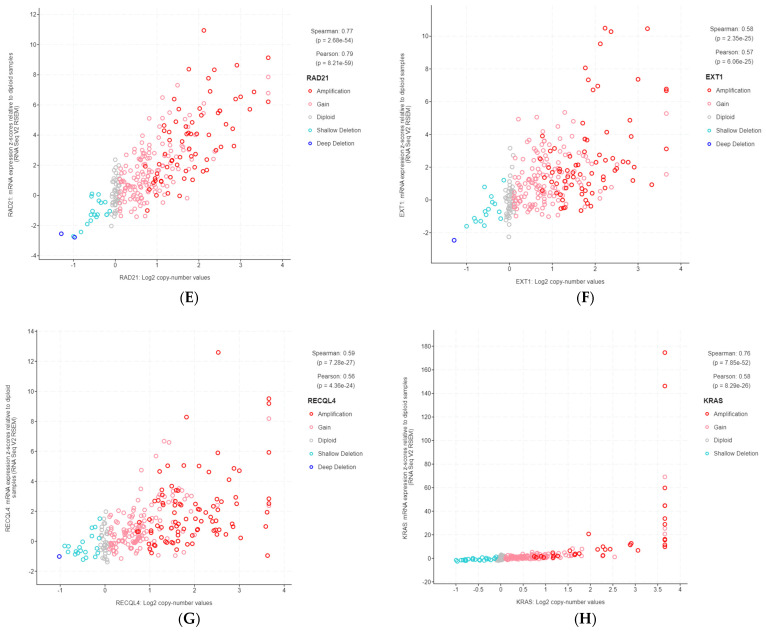

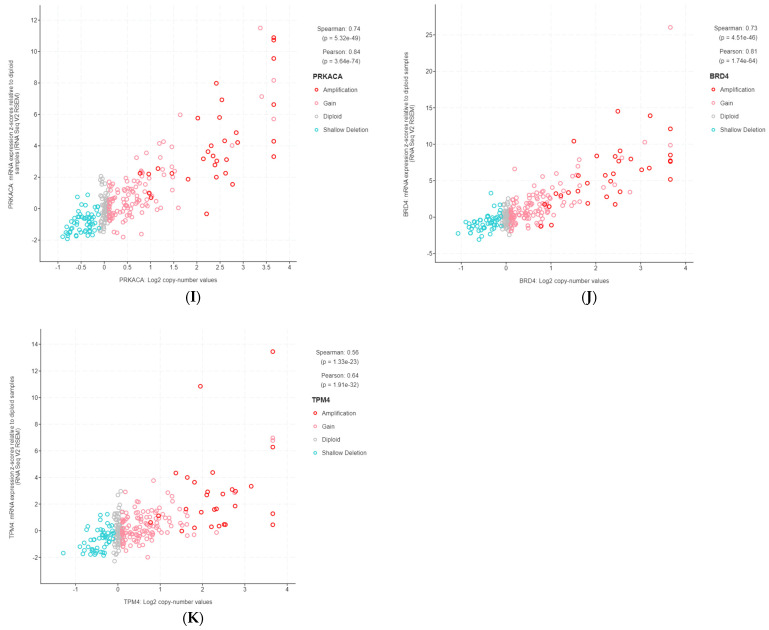
Gene copy number and mRNA expression correlation scatterplots from cBioPortal. (**A**) *TBL1XR1*; (**B**) *PIK3CA*; (**C**) *UBR5*; (**D**) *EIF3E*; (**E**) *RAD21*; (**F**) *EXT1*; (**G**) *RECQL4*; (**H**) *KRAS*; (**I**) *PRKACA*; (**J**) *BRD4*; (**K**) *TPM4*.

The 11 genes were *TBL1XR1* (r = 0.59), *PIK3CA* (r = 0.58), *UBR5* (r = 0.65), *EIF3E* (r = 0.59), *RAD21* (r = 0.79), *EXT1* (r = 0.57), *RECQL4* (r = 0.56), *KRAS* (r = 0.58), *PRKACA* (r = 0.84), *BRD4* (r = 0.81), and *TPM4* (r = 0.64) (Figure 3A–K). All of these genes were located on genomic segments that were amplified. Furthermore, several of these genes were located on the same chromosome arm, including *TBL1XR1* and *PIK3CA* on 3q; *UBR5*, *EIF3E*, *RAD21*, *EXT1*, and *RECQL4* on 8q; and *PRKACA*, *BRD4,* and *TPM4* on 19p (Table 1, Figure 2).

### 3.3. Association of Highly Correlated, Cancer-Related Genes with Clinicopathological Parameters

#### 3.3.1. Association with Age at Diagnosis

Among the profiled samples, 94 patients were younger than 55 years when diagnosed, and 168 were older than 55 years. The frequencies of gene amplifications of *EIF3E*, *RAD21*, *EXT1,* and *RECQL4* were significantly higher in those younger than 55 years. For *EIF3E*, 31 (33%) younger patients had gene amplification, whereas 20 (12%) older individuals had gene amplification (*p* = 7.077 × 10^−5^). The frequency of *RAD21* amplification in younger patients was 38% (*n* = 36) compared to 17% (n = 29) of older patients (*p* = 0.0003). For *EXT1*, younger and older patients had an amplification frequency of 41% (n = 39) and 19% (*n* = 32), respectively (*p* = 0.0001). The frequency of *RECQL4* amplification was 48% (n = 45) in younger patients and 24% (*n* = 41) in older patients (*p* = 0.0002). All other genes did not show a statistically significant association (Table 2 and Table S4).

#### 3.3.2. Association with Overall and Progression-Free Survival

**Figure 4:**
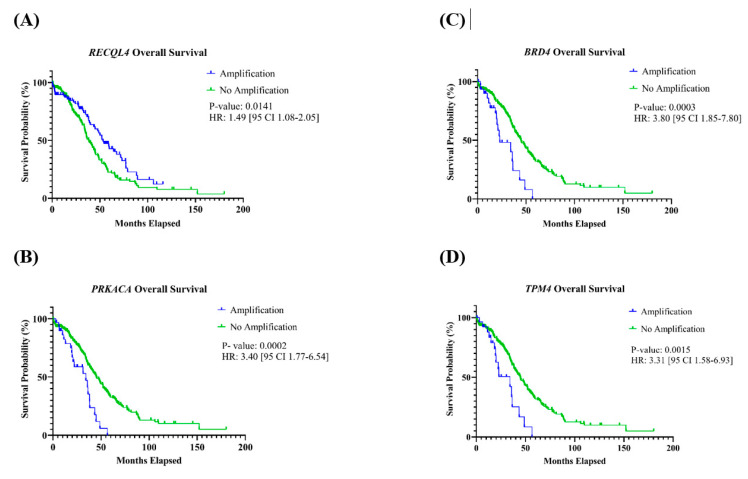
Kaplan–Meier curves of genes with an association with overall survival. Patients with amplification of the gene (**A**) *RECQL4* had a significantly better OS, and (**B**) *PRKACA*, (**C**) *BRD4*, and (**D**) *TPM4* had a significantly shorter OS.

Patients with tumors with amplification of *PRKACA*, *BRD4*, or *TPM4* found on chromosome 19 had a significantly shorter OS (Table 2). For *PRKACA*, the HR was 3.40 [95% CI: 1.77–6.54] (*p* = 0.0002); *BRD4* had an HR of 3.80 [95% CI: 1.85–7.80] (*p* = 0.0003); and the HR for *TPM4* was 3.31 [95% CI: 1.58–6.93] (*p* = 0.0015) (Figure 4A–C). Additionally, patients with tumors containing an amplification of *RECQL4*, located on chromosome 8, had a significantly better OS compared to those who did not have this gene amplification, with an HR of 1.49 [95% CI 1.08–2.05] (*p* = 0.0141) (Figure 4D). For OS, associations with other genes were not observed, and none of the 11 genes had an association with PFS (Table 2, Figures S1 and S2).

#### 3.3.3. Association with Disease Stage

There was no association between disease stage and the 11 highly correlated, Tier 1 CGC-COSMIC genes (Table 2 and Table S5).

## 4. Discussion

In this study, our primary aim was to investigate correlations between recurrent SCNA and gene expression levels within a TCGA HGOSC cohort, with a focus on identifying cancer-related genes. We identified 11 known oncogenes with both amplification and overexpression in HGOSC. The findings related to *PIK3CA*, *RAD21*, *RECQL4*, and *BRD4* align with prior studies, suggesting that overexpression of these genes is a result of amplification in HGOSC [22,30,31,32]. To our knowledge, while overexpression due to amplification in genes such as *TBL1XR1*, *PRKACA*, and *KRAS* has not been linked specifically to HGOSC, it has been reported in other gynecological cancers [33,34,35]. Moreover, *UBR5*, *EIF3E*, *EXT1,* and *TPM4* have not been previously associated with either HGOSC or other gynecological cancers in terms of copy number changes and mRNA expression level alterations. While amplifications of *MYC* and *MECOM* were observed in over 20% of tumor samples, our results did not indicate a significant correlation between gene amplification and overexpression for these genes (*MECOM*: r = 0.27; *MYC*: r = 0.40).

A subsequent aim of this study was to assess relationships between the 11 identified highly correlated, cancer-related genes and various clinicopathological parameters. Notably, several genes located at recurrent SCNA segments 8q22.2-q23.2, 8q23.3-q24.23, 8q24.23-q24.3, and 19p13.13-p13.11 yielded significant findings. The 11.55 Mb recurrent SCNA at 8q22.2-q23.2 (chr8: 98426957–109975771) encompasses two highly correlated, Tier 1 CGC-COSMIC genes: *UBR5* and *EIF3E*. While *UBR5* showed no association with any clinicopathological parameters, the frequency of *EIF3E* amplifications was linked to a younger patient age at diagnosis. Notably, the overexpression of *EIF3E* due to amplification has never been previously linked to gynecological cancers. *EIF3E* is a component of the eukaryotic initiation factor 3 complex, which is essential for initiating protein synthesis and has been associated with various cancers [36]. Amplifications of *EIF3E* have been linked to oral and colon cancers; both tumor development and a poorer prognosis have been associated with EIF3E overexpression in affected patients [37,38]. Further investigations into the amplification and overexpression of EIF3E are essential to better understand its potential oncogenic role in HGOSC.

The 23.42 Mb recurrent SCNA spanning 8q23.3-q24.23 (chr8: 112222927–135647610) encompasses two highly correlated, cancer-related genes: *RAD21* and *EXT1*. Both genes exhibited more frequent amplifications in younger patients. *RAD21* encodes RAD21 protein, an integral part of the cohesion complex vital for sister chromatid cohesion and separation, DNA damage repair, and transcription regulation [39]. While Deng et al. [30] found a robust correlation between RAD21 amplification and overexpression, as well as an association with poorer OS and PFS, our study did not corroborate these findings (OS: *p* = 0.1463; PFS: *p* = 0.3300). As for *EXT1*, although it has not been linked to gynecological cancers, its overexpression due to gene amplification has been observed in hepatocellular carcinoma [40]. Further investigations into *EXT1* could provide insights into a potential role in HGOSC.

The 6.43 Mb recurrent SCNA spanning 8q24.23-q24.3 (chr8: 138130022–145056030) features the highly correlated known cancer gene, *RECQL4*. The amplification of *RECQL4* has been linked to a younger patient age at diagnosis and improved OS compared with patients whose tumors do not have gene amplification. *RECQL4* encodes a protein belonging to the RecQ helicase family, crucial for maintaining genomic stability, and has been implicated in various cancers, including HGOSC [41]. Guo et al. [22] found overexpression of *RECQL4* due to amplification in HGOSC and associated overexpression with poorer patient OS and PFS. However, our findings diverge, indicating significantly better OS for individuals with tumors showing *RECQL4* amplification, with no discernible association with PFS. The potential prognostic impact of *RECQL4*, especially for younger individuals with HGSOT, warrants further analysis.

The 2.74 Mb recurrent SCNA located at chr19:19p13.13-p13.11 encompasses three highly correlated, cancer-related genes: *PRKACA*, *BRD4*, and *TPM4*. Individuals with tumors with amplifications of these genes had poorer OS. *BRD4*, a well-established oncogene linked to HGOSC, had a strong correlation between amplification and overexpression, as has been shown by others. Additionally, elevated mRNA levels were associated with a worse OS [10,31]. *PRKACA* encodes the PRK catalytic subunit alpha isoform, and its abnormalities have been linked to various diseases [42]. A translocation involving PRKACA has been identified in fibrolamellar hepatocellular carcinoma [43]. Meanwhile, *TPM4* encodes a protein belonging to the tropomyosin family, playing a role in muscle contraction and in maintaining the stability and function of the non-muscle cell cytoskeleton [44]. *TPM4* has been associated with cancers such as hepatocellular carcinoma and glioma [45,46]. Further research into *PRKACA* and *TPM4* is essential to determine their potential impact on HGOSC pathogenesis and to ascertain if *BRD4* is a primary gene influencing HGOSC.

This study has several limitations. First, only 272 of the 585 available OSC profiled samples met our inclusion criteria for this study. This reduction might have inadvertently included or excluded genes that could have been present or absent in the entire cohort. However, a sample size of 272 remains substantial. Secondly, the identified candidate cancer genes might be specific to this TCGA HGOSC cohort. Validating these results with another cohort or new HGOSC tumor samples in subsequent studies would reinforce our findings. Third, the TCGA is a multi-institutional retrospective study focused on characterizing somatic genetic aberrations and lacks consistent treatment information, thereby limiting the scope of prognosis-related findings. A clinical trial might offer a more comprehensive assessment of prognostic implications. Fourth, our decision to use a cutoff value of 10% for gene recurrent SCNA frequency and a Pearson correlation coefficient of r ≥ 0.55 is somewhat arbitrary, although a similar approach was used in a study on endometrial serous carcinoma [34]. Another limitation is the exclusion of protein data, which could offer deeper insights into genes with recurrent SCNA that are abnormally expressed and influence HGOSC pathogenesis. Sixth, while we utilized the Tier 1 CGC-COSMIC list for cancer-related genes, other databases like OncoKB and myCancerGenome offer valuable gene-cancer associations [47,48]. Incorporating these databases in future studies might help identify additional oncogenes and tumor suppressor genes. Lastly, functional studies on the candidate oncogenes could elucidate the role these alterations play in the development and progression of HGOSC.

## 5. Conclusions

The results indicate that the overexpression of key oncogenes in HGOSC, such as *BRD4*, *KRAS,* and *PIK3CA,* are driven by somatic copy number alterations. However, amplifications do not invariably result in gene expression alterations, as evidenced by the high frequency of *MECOM* and *MYC* amplifications. Gene amplifications of *EIF3E*, *RAD21*, *EXT1,* and *RECQL4* are more prevalent in HGOSC in younger patients, and *RECQL4*, *PRKACA*, *BRD4*, and *TPM4* have prognostic significance. Further research is warranted to comprehensively understand the influence of gene copy number variations on HGOSC pathogenesis.

## Figures and Tables

**Table 1:** Genomic blocks of recurrent SCNAs in high-grade ovarian serous carcinoma.

Cytoband	GRCh38 Coordinates	Size (Mb)	CNV	Tier 1 CGC-COSMIC Genes
1p34.3-p34.2	chr1:38838197–41242306	2.40 Mb	AMP	
3q35.2-q29	chr3:155290226–198222513	42.93 Mb	AMP	*MECOM*, *TBL1XR1* *, *PIK3CA* *, *SOX2*, *MAP3K13*, *ETV5*, *EIF4A2*, *BCL6*, *LPP*, *TP63*
5q11.2-q12.1	chr5:58969037–60488065	1.52 Mb	DEL	
8p23.3-p23.2	chr8:166085–4994914	4.83 Mb	DEL	
8q22.2-q23.2	chr8:98426957–109975771	11.55 Mb	AMP	*UBR5* *, *RSPO2*, *EIF3E* *
8q23.3-q24.23	chr8:112222927–135647610	23.42 Mb	AMP	*RAD21* *, *EXT1* *, *MYC*, *NDRG1*
8q24.23-q24.3	chr8:138130022–145056030	6.93 Mb	AMP	*RECQL4* *
11q14.1-q14.1	chr11:77589952–79441030	1.85 Mb	AMP	
12p12.1-p11.23	chr12:23529503–26833194	3.30 Mb	AMP	*KRAS* *
19p13.13-p13.11	chr19:13731751–16471970	2.74 Mb	AMP	*PRKACA* *, *DNAJB1*, *BRD4* *, *TPM4* *
19p12-p12	chr19:23914885–24163447	0.25 Mb	AMP	
19q12-q13.11	chr19:28965130–34677159	5.71 Mb	AMP	*CCNE1*
19q13.2-q13.2	chr19:38303557–39934634	1.63 Mb	AMP	
22q13.32-q13.33	chr22:48489552–50799637	2.31 Mb	DEL	

* Genes in bold format have a Pearson correlation coefficient r ≥ 0.55.

**Table 2:** Summary of highly correlated Tier 1 CGC-COSMIC genes with clinicopathological parameters.

Tier 1 CGC-COSMIC Gene	Association with Stage	Association with Age	Association with PFS	Association with OS
*TBL1XR1*	*p* = 1.000	*p* = 0.2251	*p* = 0.1131	*p* = 0.1011
*PIK3CA*	*p* = 1.000	*p* = 0.0896	*p* = 0.3638	*p* = 0.2521
*UBR5*	*p* = 0.4816	*p* = 0.0665	*p* = 0.1242	*p* = 0.3576
*EIF3E*	*p* = 0.2104	*p* = 7.077 × 10^−5^ *	*p* = 0.7110	*p* = 0.7091
*RAD21*	*p* = 0.7707	*p* = 0.0003 *	*p* = 0.3300	*p* = 0.1463
*EXT1*	*p* = 0.5722	*p* = 0.0001 *	*p* = 0.8829	*p* = 0.3558
*RECQL4*	*p* = 0.5947	*p* = 0.0002 *	*p* = 0.2713	*p* = 0.0141 *
*KRAS*	*p* = 1.000	*p* = 0.5480	*p* = 0.4719	*p* = 0.1313
*PRKACA*	*p* = 0.7026	*p* = 0.1137	*p* = 0.4412	*p* = 0.0002 *
*BRD4*	*p* = 0.2362	*p* = 0.4320	*p* = 0.7229	*p* = 0.0003 *
*TPM4*	*p* = 1.000	*p* = 0.5322	*p* = 0.7904	*p* = 0.0015 *

* Genes in bold were statistically significant (*p* < 0.05).

## Data Availability

Data are contained within this article or Supplementary Material.

## Supplementary Materials

The following supporting information can be downloaded at: https://www.mdpi.com/article/10.3390/life13112192/s1, Figure S1: Overall Survival Kaplan-Meier Curves for 11 Highly Correlated Tier 1 CGC-COSMIC Genes A. *TBL1XR1*; B. *PIK3CA*; C. *UBR5*; D. *EIF3E*; E. *RAD21*; F. *EXT1*; G. *RECQL4*; H. *KRAS*; I. *PRKACA*; J. *BRD4*; K. *TPM4*; Figure S2: Progression Free Survival Kaplan-Meier Curves for 11 Highly Correlated Tier 1 CGC-COSMIC Genes. A. *TBL1XR1*; B. *PIK3CA*; C. *UBR5*; D. *EIF3E*; E. *RAD21*; F. *EXT1*; G. *RECQL4*; H. *KRAS*; I. *PRKACA*; J. *BRD4*; K. *TPM4*; Table S1: Clinical data for 272 profiled samples; Table S2: Genes in genomic blocks of recurrent SCNA in high-grade ovarian serous carcinoma; Table S3: All Tier 1 CGC-COSMIC genes; Table S4: Contingency Tables for Association with Age at Diagnosis; Table S5: Contingency Tables for Association with Disease Stage.
