# Supplementary material for: Recurrent Somatic Copy Number Alterations and Their Association with Oncogene Expression Levels in High-Grade Ovarian Serous Carcinoma
Source: Life (Basel). 2023 Nov 10;13(11):2192. doi: 10.3390/life13112192 (PMC10672014; doi:10.3390/life13112192)

Figure S1. Overall Survival Kaplan-Meier Curves for 11 Highly Correlated Tier 1 CGC-COSMIC Genes A. *TBL1XR1*; B. *PIK3CA*; C. *UBR5*; D. *EIF3E*; E. *RAD21*; F. *EXT1*; G. *RECQL4*; H. *KRAS*; I. *PRKACA*; J. *BRD4*; K. *TPM4*

(A)

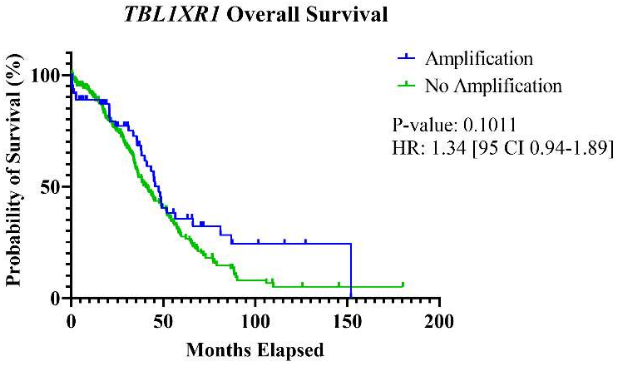

(B)

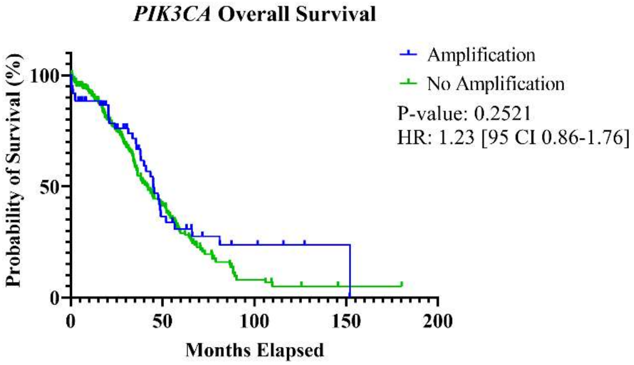

(C)

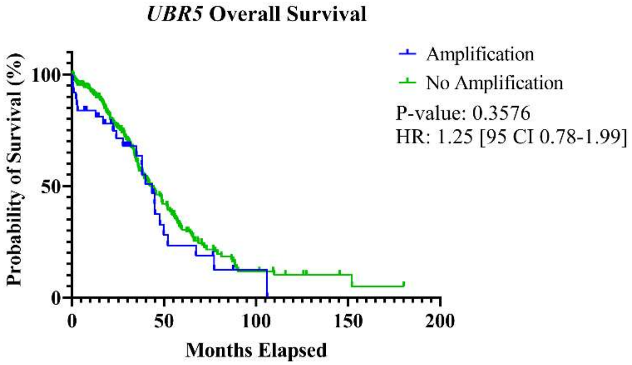

(D)

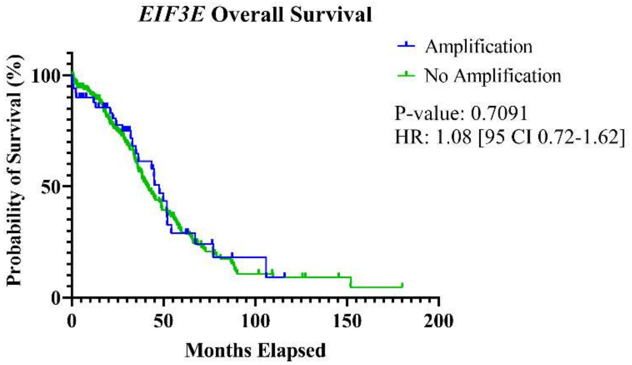

Figure S1 (Continued)

(E)

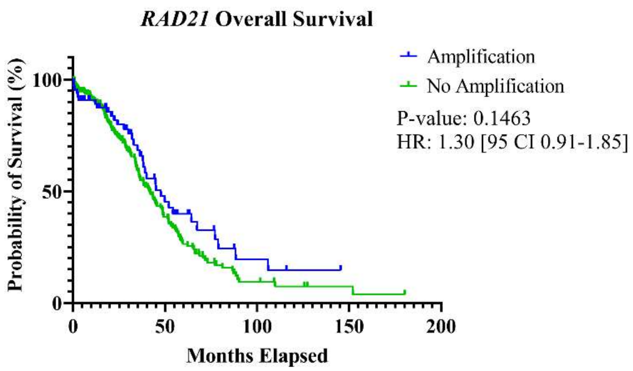

(G)

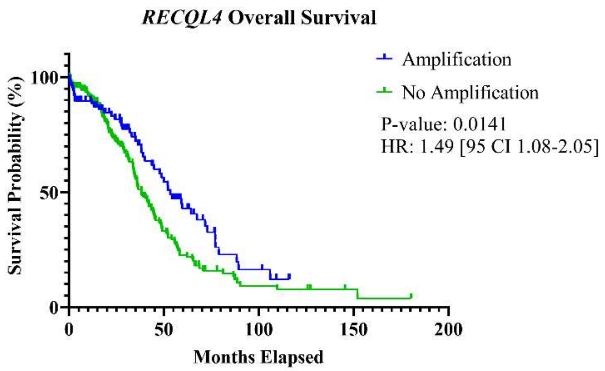

(F)

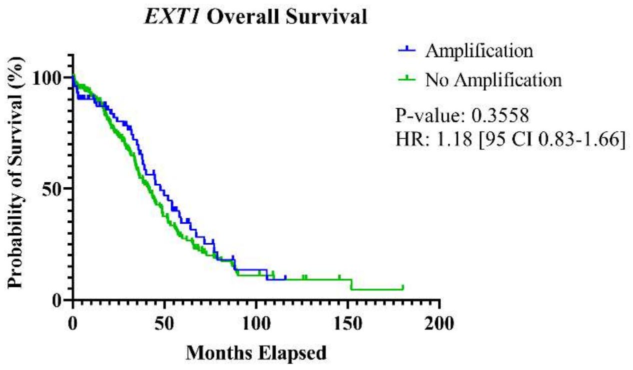

(H)

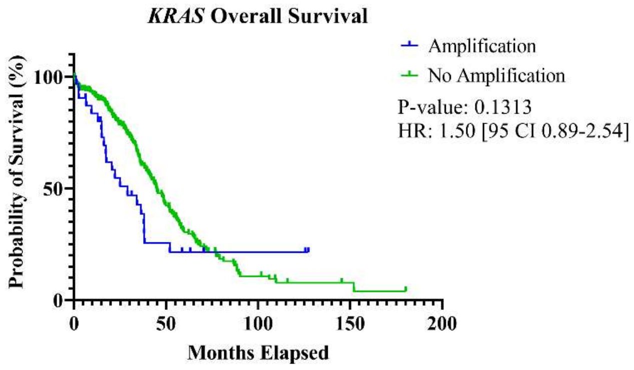

Figure S1 (Continued)

(I)

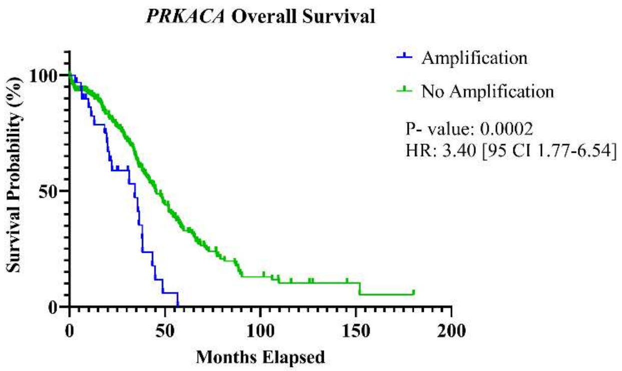

(K)

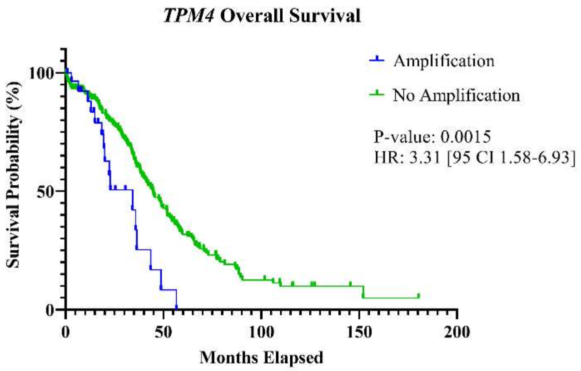

(J)

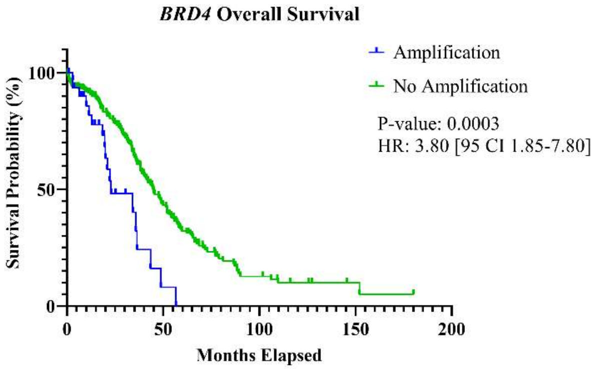

Figure S2. Progression Free Survival Kaplan-Meier Curves for 11 Highly Correlated Tier 1 CGC-COSMIC Genes. A. *TBL1XR1*; B. *PIK3CA*; C. *UBR5*; D. *EIF3E*; E. *RAD21*; F. *EXT1*; G. *RECQL4*; H. *KRAS*; I. *PRKACA*; J. *BRD4*; K. *TPM4*

(A)

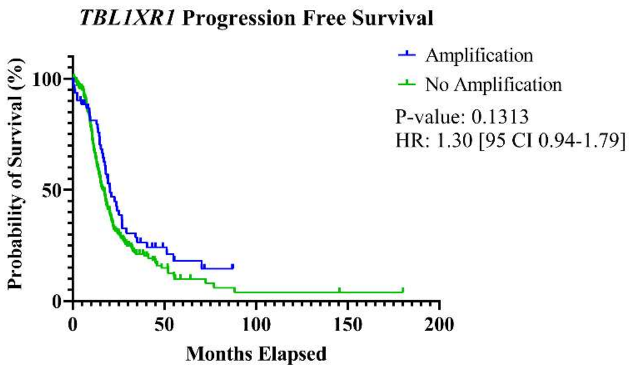

(B)

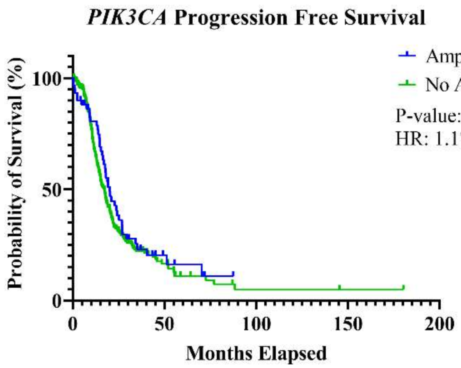

(C)

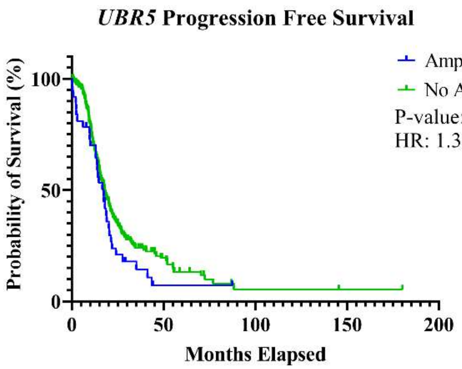

(D)

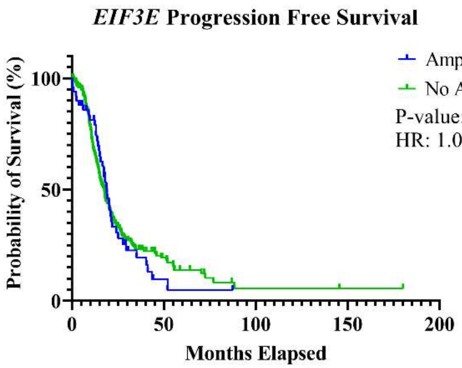

Figure S2 (Continued)

(E)

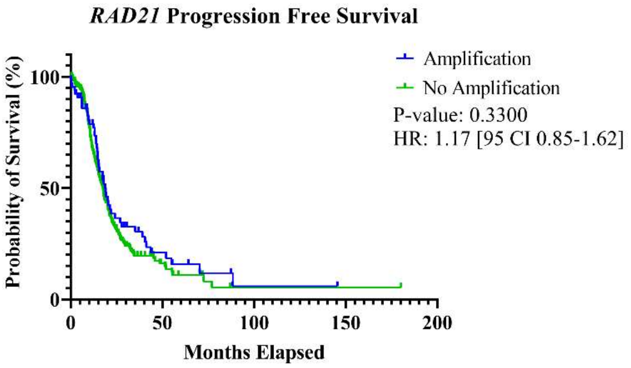

(G)

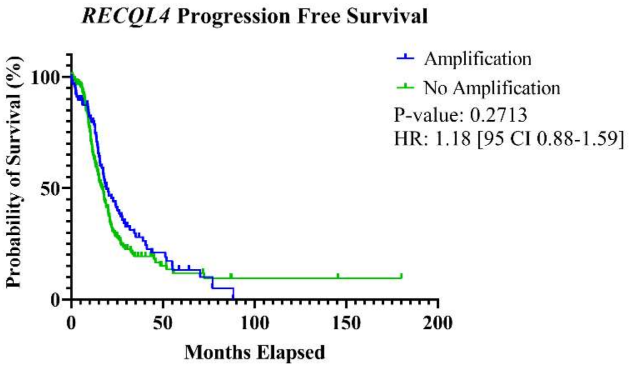

(F)

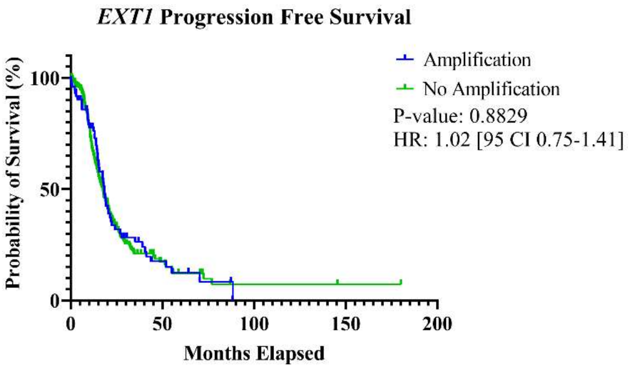

(H)

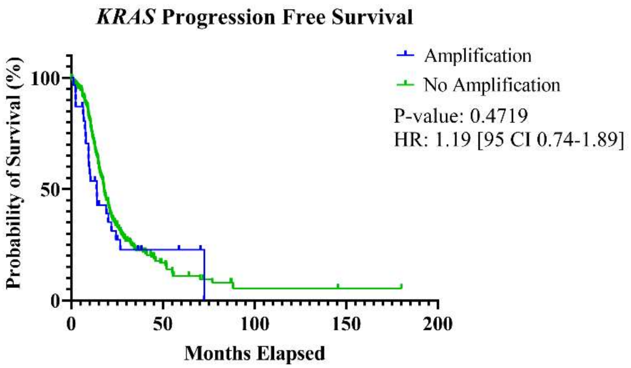

Figure S2 (Continued)

(I)

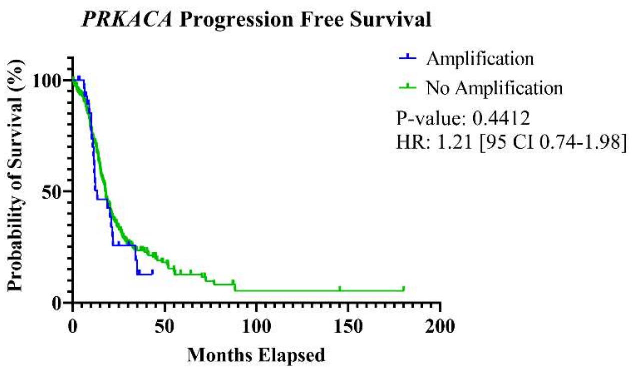

(K)

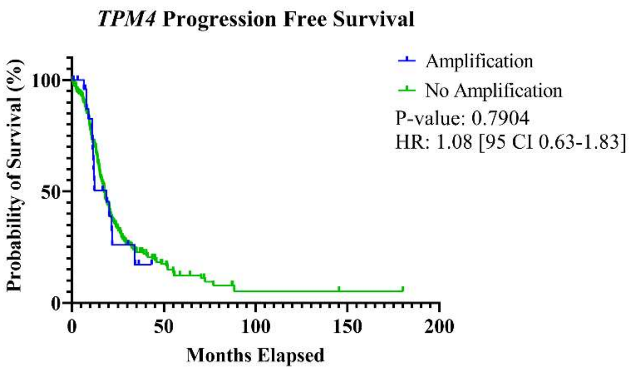

(J)

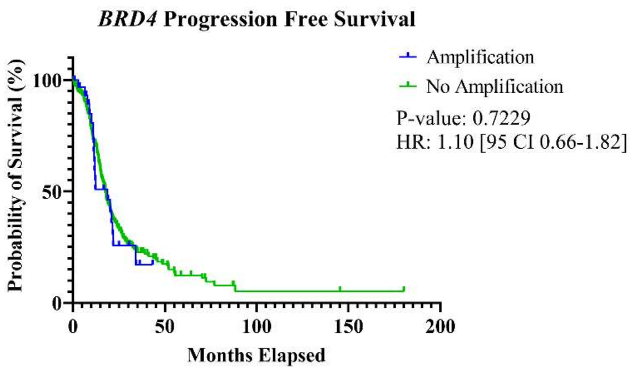

Supplement: Supplementary file 1 [file life-13-02192-s001.zip › FigureS1_S2Esplen.pdf]
