# Supplementary material for: Recurrent Somatic Copy Number Alterations and Their Association with Oncogene Expression Levels in High-Grade Ovarian Serous Carcinoma
Source: Life (Basel). 2023 Nov 10;13(11):2192. doi: 10.3390/life13112192 (PMC10672014; doi:10.3390/life13112192)
Supplement: Supplementary file 1 [file life-13-02192-s001.zip › Supplementary Table 4-5.pdf]

Table S4: Contingency Tables for Association with Age at Diagnosis

.

A. *TBL1XR1*; B. *PIK3CA*; C. *UBR5*; D. *EIF3E*; E. *RAD21*; F. *EXT1*; G. *RECQL4*; H. *KRAS*; I. *PRKACA*; J. *BRD4*; K. *TPM4*

(A) *TBL1XR1* (p = 0.2251)

	**Amplified**	**Not Amplified**	**Total**
**< 55**	26	68	94
**≥ 55**	35	133	168
**Total**	61	201	262

(B) *PIK3CA* (p = 0.0896)

	**Amplified**	**Not Amplified**	**Total**
**< 55**	27	67	94
**≥ 55**	32	136	168
**Total**	59	203	262

C) *UBR5* (p = 0.0665)

	**Amplified**	**Not Amplified**	**Total**
**< 55**	19	75	94
**≥ 55**	19	149	168
**Total**	38	224	262

(D) *EIF3E* (p = 7.077e-05)

	**Amplified**	**Not Amplified**	**Total**
**< 55**	31	63	94
**≥ 55**	20	148	168
**Total**	51	211	262

(E) *RAD21* (p = 0.0003)

	**Amplified**	**Not Amplified**	**Total**
**< 55**	36	58	94
**≥ 55**	29	139	168
**Total**	65	197	262

(F) *EXT1* (p = 0.0001)

	**Amplified**	**Not Amplified**	**Total**
**< 55**	39	55	94
**≥ 55**	32	136	168
**Total**	71	191	262

G) *RECQL4* (p = 0.0002)

	**Amplified**	**Not Amplified**	**Total**
**< 55**	45	49	94
**≥ 55**	41	127	168
**Total**	86	176	262

(H) *KRAS* (p = 0.5480)

	**Amplified**	**Not Amplified**	**Total**
**< 55**	9	85	94
**≥ 55**	21	147	168
**Total**	30	232	262

I) *PRKACA* (p = 0.1137)

	**Amplified**	**Not Amplified**	**Total**
**< 55**	7	87	94
**≥ 55**	24	144	168
**Total**	31	231	262

(J) *BRD4* (p = 0.4320)

	**Amplified**	**Not Amplified**	**Total**
**< 55**	9	85	94
**≥ 55**	23	145	168
**Total**	32	230	262

K) *TPM4* (p = 0.5322)

	**Amplified**	**Not Amplified**	**Total**
**< 55**	8	86	94
**≥ 55**	20	148	168
**Total**	28	234	262

Table S5: Contingency Tables for Association with Disease Stage.

A. *TBL1XR1*; B. *PIK3CA*; C. *UBR5*; D. *EIF3E*; E. *RAD21*; F. *EXT1*; G. *RECQL4*; H. *KRAS*; I. *PRKACA*; J. *BRD4*; K. *TPM4*

(A) *TBL1XR1* (p = 1.000)

	**Amplified**	**Not Amplified**	**Total**
**Early Stage**	1	16	17
**Late Stage**	27	226	253
**Total**	28	242	270

(B) *PIK3CA* (p = 1.000)

	**Amplified**	**Not Amplified**	**Total**
**Early Stage**	4	13	17
**Late Stage**	56	197	253
**Total**	60	210	270

(C) *UBR5* (p = 0.4816)

	**Amplified**	**Not Amplified**	**Total**
**Early Stage**	1	16	17
**Late Stage**	37	216	253
**Total**	38	232	270

(D) *EIF3E* (p = 0.2104)

	**Amplified**	**Not Amplified**	**Total**
**Early Stage**	1	16	17
**Late Stage**	50	203	253
**Total**	51	219	270

(E) *RAD21* (p = 0.7707)

	**Amplified**	**Not Amplified**	**Total**
**Early Stage**	3	14	17
**Late Stage**	63	190	253
**Total**	66	204	270

(F) *EXT1* (p = 0.5722)

	**Amplified**	**Not Amplified**	**Total**
**Early Stage**	3	14	17
**Late Stage**	69	184	253
**Total**	72	198	270

(G) *RECQL4* (p = 0.5947)

	Amplified	Not Amplified	Total
**Early Stage**	4	13	17
**Late Stage**	85	168	253
**Total**	89	181	270

(H) *KRAS* (p = 1.000)

	Amplified	Not Amplified	Total
**Early Stage**	2	15	17
**Late Stage**	29	224	253
**Total**	31	239	270

(I) *PRKACA* (p = 0.7026)

	Amplified	Not Amplified	Total
**Early Stage**	1	16	17
**Late Stage**	30	223	253
**Total**	31	239	270

(J) *BRD4* (p = 0.2362)

	**Amplified**	**Not Amplified**	**Total**
**Early Stage**	0	17	17
**Late Stage**	32	221	253
**Total**	32	238	270
